# DectICO: an alignment-free supervised metagenomic classification method based on feature extraction and dynamic selection

**DOI:** 10.1186/s12859-015-0753-3

**Published:** 2015-10-07

**Authors:** Xiao Ding, Fudong Cheng, Changchang Cao, Xiao Sun

**Affiliations:** State Key Laboratory of Bioelectronics, School of Biological Science and Medical Engineering, Southeast University, Nanjing, 210096 China

**Keywords:** Alignment-free, Metagenome, Classification, Sequence feature, Feature selection

## Abstract

**Background:**

Continual progress in next-generation sequencing allows for generating increasingly large metagenomes which are over time or space. Comparing and classifying the metagenomes with different microbial communities is critical. Alignment-free supervised classification is important for discriminating between the multifarious components of metagenomic samples, because it can be accomplished independently of known microbial genomes.

**Results:**

We propose an alignment-free supervised metagenomic classification method called DectICO. The intrinsic correlation of oligonucleotides provides the feature set, which is selected dynamically using a kernel partial least squares algorithm, and the feature matrices extracted with this set are sequentially employed to train classifiers by support vector machine (SVM). We evaluated the classification performance of DectICO on three actual metagenomic sequencing datasets, two containing deep sequencing metagenomes and one of low coverage. Validation results show that DectICO is powerful, performs well based on long oligonucleotides (i.e., 6-mer to 8-mer), and is more stable and generalized than a sequence-composition-based method. The classifiers trained by our method are more accurate than non-dynamic feature selection methods and a recently published recursive-SVM-based classification approach.

**Conclusions:**

The alignment-free supervised classification method DectICO can accurately classify metagenomic samples without dependence on known microbial genomes. Selecting the ICO dynamically offers better stability and generality compared with sequence-composition-based classification algorithms. Our proposed method provides new insights in metagenomic sample classification.

**Electronic supplementary material:**

The online version of this article (doi:10.1186/s12859-015-0753-3) contains supplementary material, which is available to authorized users.

## Background

Metagenomics has revolutionized microbiology by allowing a cultivation-independent assessment and exploitation of microbial communities present in complex ecosystems [1, 2]. Advances in next-generation sequencing technologies, coupled with new bioinformatics developments, have facilitated rapid and high-throughput metagenomic sequencing, promoting the development of metagenomics. Consequently, thousands of metagenomic projects have been completed, and have provided valuable insight into many different microbial communities. For example, among many others, metagenomes from the Sargasso Sea [3], the human gut microbiome [4], and an acidic mine drainage biofilm [5] have all been sequenced. The human body is inhabited by at least ten times more microbes than the number of human cells in the body [6], and these various microbes play fundamental roles in human health and disease. Microbiomes are involved in human metabolism, nutrition, immune system development, and a wide range of other functions [7–9]. Therefore, more and more research has focused on human microbiomes. For example, the Human Microbiome Project [10] was funded by the National Institutes of Health (NIH), resulting in a broad range of quality-controlled resources of high-throughput metagenomic data available to the scientific community. This large-scale source of sequencing data corresponds to various metagenomic samples, including different individual health states and/or different parts of the human body. Therefore, comparing and classifying microbial samples becomes increasing important for studying microbiomes.

Before the emergence of the sequencing technologies, the single-gene rRNA surveys had been an important form of culture-independent genomics [11]. And now sequencing ribosomal RNA subunits, especially 16S rRNA, has provided valuable insights into the diversity of thousands of uncultured microbial samples from various environments. The global diversity of metagenomic samples can be divided into three components: alpha diversity, the richness of taxa in a single sample; beta diversity, the differentiation between samples or communities; and gamma diversity, the differentiation between geographic regions [12–15]. Beta diversity can be analyzed by comparing microbial catalogs between different samples. Sequenced 16S rRNA gene fragments are first clustered into different operational taxonomic units (OTUs) in 16S rRNA-based research, then each OTU is assigned to a particular taxon by comparison against known 16S rRNA databases (e.g., RDP, Greengene and SILVA [16–18]). Many 16S rRNA-based analytical pipelines have recently and widely been adopted, including mothur [19], Qiime [20] and the RDP pipeline [17], among many others. However, 16S rRNA-based studies can only provide limited information, especially regarding the functional analysis of microbiomes. In contrast, whole metagenome shotgun sequencing (WMGS) can provide more complete information on microbial communities. WMGS provides not only the taxonomic profile of the community, but also the full genetic information of all the microbes in the community sampled, allowing a more thorough understanding of the interactions between microbes and the environment in which they exist.

Approaches for comparing and classifying metagenomic samples include alignment-based and alignment-free methods. In alignment-based methods raw reads are first assembled into contigs. Then a taxonomic profile and a catalog of genes are obtained for each sample by mapping these contigs to databases of microbial genomes and genes (e.g., the NCBI nr database, the KEGG database, and the COG database). Differences between metagenomic samples are calculated based on profiles of taxonomy and function. Finally, the samples can be assigned into informative classes by employing supervised classification methods. Alternatively, the samples can be clustered using unsupervised machine learning methods to find intrinsic clusters [21–24]. However, the limitations of alignment-based methods are obvious. A sparsity of known microbial genomes and genes is the primary bottleneck, results in that a large number of fragments of metagenomic data can’t map to the known database [25, 26]; In addition, the alignment of a huge number of contigs is computationally intensive and time-consuming. Therefore, alignment-free methods are a promising approach for analyzing such huge metagenomic datasets. Alignment-free methods are based on one or more sequence features, combined with supervised or unsupervised machine learning algorithms, independent of reference databases, unlike alignment-based methods. Previous observations have determined that tetra-nucleotide frequency composition is an optimal feature for discriminating species taxonomy [27]. Sequence composition was first used as an alignment-free metagenomic binning method for clustering individual metagenomic fragments [28–31]. A novel sequence feature, the intrinsic correlation of oligonucleotides (ICO), was proposed, and has proven more powerful for distinguishing microbial species by extracting more significant differences between genomic sequences, than sequence-composition-based feature methods in our previous researches [32]. Differences between the sequence feature vectors that represent metagenomic samples can distinguish the metagenomic samples. Previous studies have proven that alignment-free unsupervised classification methods can reveal dissimilarities between metagenomic samples, and cluster those samples into reasonable classes [33–35]. However, these methods can only discover major intrinsic clustering relations among the compared samples, and are sometimes invalid when the classes are predefined (See Additional file 1: Figure S1). For example, we are interested in distinguishing human gut metagenomic samples associated with inflammatory bowel disease (IBD) from healthy human gut metagenomes. Unfortunately, some sample subjects also suffer from type 2 diabetes (T2D), with a similar frequency as the IBD disease samples. An unsupervised algorithm-based classification method may incorrectly cluster samples into a T2D group and a healthy group. In other words, the class of interest (here, the IBD samples) may not dominate the assortment of data and, therefore, may not be revealed as a separate group in an unsupervised comparison. Supervised classification methods are more suitable in this situation. A classifier can be built with a specific sequence feature, to classify samples into predefined classes, using samples with known classification labels as training sets.

In this paper, we propose DectICO, an alignment-free supervised algorithm that dynamically selects the ICO set using kernel partial least squares (*kpls*) [36], for classifying metagenomic samples, focusing on the beta diversity of metagenomic samples. A given ICO feature set based on long oligonucleotides usually has a high dimensionality, resulting in an inaccurate supervised classification, owing to an excess of noise components in the high-dimension feature set. Furthermore, a high-dimension feature set also increases computational complexity. Therefore, we refine the entire ICO feature set in our method. DectICO is a supervised algorithm which uses a set of completely labeled samples to train a classifier, and then classifies the unlabeled samples.

We evaluated the performance of our method on three groups of actual metagenomic sequencing data: two containing deep sequencing metagenomes, and one metagenome of low sequencing depth. We demonstrate that our method performs better than a sequence-composition-based method, especially based on long oligonucleotides, not only for the deep sequencing metagenomic datasets, but also for the low coverage dataset. The sequence-composition-based method employs sequence composition instead of the ICO, but uses the dynamic *kpls* feature selection as same as DectICO. Additionally, we demonstrate that the dynamic *kpls* feature selection technique performs better than the non-dynamic *kpls* feature selection approach. Our experimental results also demonstrate that DectICO has better stability and generality than the recursive support vector machine (RSVM)-based classification algorithm [37].

## Methods

### The algorithm of DectICO

**Fig. 1:**
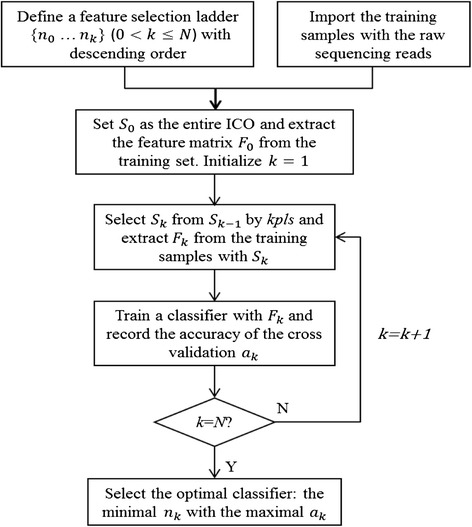
DectICO’s algorithm scheme

We propose for DectICO to select an optimum feature set from all ICO components dynamically, and to train classifiers with feature matrices extracted from those feature sets. The algorithm scheme is described in Fig. 1. We let *n*_*k*_(0 ≤ *k* ≤ *N*) stand for the size of the feature set to be selected in round *k*, with descending order (*n*_0_ > *n*_1_ > … > *n*_*N*_), and *n*_0_ represents the size of the entire ICO. The maximal number of the round for selecting features is denoted by *N*, which is defined by users. *S*_0_ is defined as the entire feature set, and consists of all components of the ICO. For each round, the selected feature set of size *n*_*k*_ is denoted by *S*_*k*_(0 < *k* ≤ *N*), which is the subset of *S*_*k* − 1_. The feature matrix extracted from the training data with *S*_*k*_ is defined as *F*_*k*_(0 < *k* ≤ Ν) whose rows represent the feature vectors extracted from training samples. And *F*_0_ means the feature matrix extracted from training samples with *S*_0_. In addition, *a*_*k*_(0 < *k* ≤ Ν) represents the accuracies of a leave-one-out cross validation (LOOCV) of the classifier trained with *F*_*k*_ in each round.

The entire workflow of DectICO contains the following steps:

1. Input: Give a set of training samples the raw sequencing reads and a predefined feature selection ladder {*n*_0_ … *n*_*k*_}(0 < *k* ≤ Ν) of descending order.
2. Initialization: Set *S*_0_ as the entire ICO and extract the feature matrix *F*_0_ from the training set. Initialize *k* = 1.
3. Recursion: Select the feature set *S*_*k*_ from *S*_*k* − 1_ by *kpls* and extract *F*_*k*_ from the training samples with *S*_*k*_. Train a classifier with *F*_*k*_ and record the accuracy by LOOCV of *a*_*k*_. Let *k* = *k* + 1 and repeat this process until *k* = *N*.
4. Output: Select the optimal classifier with the minimal number of feature components *n*_*k*_ and the maximal accuracy by LOOCV of *a*_*k*_.

DectICO employs the *kpls* algorithm to select the entire ICO set dynamically. Therefore, the key characteristics of our algorithm are its use of the ICO and dynamic feature selection. We compared classification performance between our ICO-based and a sequence-composition-based method, as well as between dynamic and non-dynamic feature selection methods.

Non-dynamic feature selection methods select a feature set from the entire feature only once for each size of selected set. Furthermore, feature selection by *kpls* is based on the weights of all features defined by *kpls.* The weight rank of the entire feature is not necessarily suitable for each size of feature set. Dynamic feature selection overcomes this problem by selecting the entire feature many times, which updates the weight of the selected feature set in each round. DectICO is implemented in Perl and Matlab, and was built using kernelPLS [36] and Libsvm [38], it can run both on the Windows and the Linux system. Source code is available at https://github.com/dingxiao8715/DectICO. Although DectICO isn’t characterized by fast and low RAM consumed, we also gathered a statistics of the runtime and required RAM of DectICO (See Additional file 1: Table S6 ~ S9). The results indicate that DectICO has acceptable runtime and consumed RAM.

### Intrinsic correlation of oligonucleotides

The sequence feature itself is the most important element of alignment-free classification methods. There are two kinds of sequence features: sequence composition and sequence correlation. Sequence composition measures the content of different components in a DNA sequence, such as a single base or an oligonucleotide component, and it is wide used in genome analysis. However, sequence correlation represents the relationship among different components in genomes, which contains deeper information of genomes. In this study, we investigated classification performance for both the ICO and a sequence-composition-based method.

As a kind of sequence correlation, ICO represents the correlation between two consecutive parts of oligonucleotides with fixed length [32]. Given an oligonucleotide with length *k* (i.e., *k*-mer), we can separate it into two consecutive parts *i* and *j* with length *m* and *n* respectively (*m* ranges from 1 to *k*-1 and *n* = *k*-*m*). The ICO (*m*, *n*) for a genomic sequence *S* is defined as a descriptor that indicates the correlation between any consecutive part *i* and *j* within *S*. Let A and B be sets of all oligonucleotides with length *m* and *n* respectively. The counts of components in sets A and B depend on the length of *i* and *j*. For example, when we evaluate ICO (1, 3), *i* represents arbitrary single base like A, C and *j* means arbitrary trimers like ACT or GAT, all components in A are {A, C, T, G} and B contains all 64 kinds of trimers {AAA, AAC… GGG}. According to the rationale above, the ICO for a genomic sequence *S* based on the *k*-mer is a combination of *k*-1 types of ICO, i.e., ICO (1, *k*-1), ICO (2, *k*-2)…ICO (*k*-1, 1). For example, the ICO based on 4-mer contains ICO (1, 3), ICO (2, 2), and ICO (3, 1). In general, the ICO (*m*, *n*) consists of two sections: the first section describes the correlation between two consecutive oligonucleotides (or bases), namely *i* and *j*, and the second section represents the average mutual information between them. The definition for the first section follows:

$$ {f}_{ij}=\frac{p_{ij}}{p_i{p}_j} $$

where *p*_*ij*_ represents the probability of occurrence of junction between the two oligonucleotides *i* and *j*, and *p*_*i*_ and *p*_*j*_ represent the probability of occurrence of *i* and *j*, respectively, in a sequence.

The second section of the ICO vector is based on information theory. We proposed this section to help explore deeper relationships between two oligonucleotides. The definition for this section is:

$$ I(i)={\displaystyle \sum_{j\in B}{p}_{j/i}{ \log}_2\left(\frac{p_{ij}}{p_i{p}_j}\right)}\kern0.5em \left(i\in A\right) $$

where A and B are the sets of all oligonucleotides with length *m* and *n* respectively, respectively. *I*(*i*) represents the average mutual information of *i* acquired from *j. p*_*i*_, *p*_*j*_, and *p*_*ij*_ are the same as in the above equation. *p*_*j*/*i*_ represents the conditional probability of the occurrence of *j*, on the condition that *i* is fixed. The performance of distinguishing genomes with the ICO is detailed by [32].

It is noteworthy that we calculate the feature vector for each metagenomic sample, which is regarded as an integrated one, instead of extracting the feature of each read in a sample, and then computing the average feature vector.

The ICO and composition (each component represents the occurrence frequency of every oligonucleotide in a metagenomic sample) vectors are not of the same magnitude; therefore, we employ a simple normalization method described below:

$$ {v_i}^{\hbox{'}}=\frac{v_i-{v}_{\min }}{v_{\max }-{v}_{\min }}\left(i=1\dots n\right) $$

We assume the feature vector has n dimensions, and denote the original and normalized vector element by *v*_*i*_ and *v*_*i*_^'^, respectively. *v*_max_ and *v*_min_ represent the maximum and minimum value among these components, respectively.

### Kernel partial least squares

We employ the *kpls* algorithm [36] for feature selection, which was first proposed for selecting features from microarray gene expression data for cancer sample classification. *Kpls* is based on *pls* [39] and the theory of Reproducing Kernel Hilbert Space [40]. In the following, we introduce the basic algorithm of *kpls* briefly.

*Pls* is one of a broad class of methods for modeling relations between sets of observed features by means of latent variables called components [41]. In order to describe the algorithm conveniently, we denote *X* as a data matrix with *N* samples and $\underline{y}$ as the class vector of the samples. The basic goal of *pls* is to obtain a low dimensional approximation of a data matrix *X* such that the approximation will be as close as possible to a given vector $\underline{y}$. Namely, *pls* seeks a *k* × 1 vector $\underline{w}$ satisfying $\left\Vert \underline{w}\right\Vert =1$ and that maximizes $\operatorname{cov}\left(X\underline{w},\underline{y}\right)$. $X\underline{w}$ is denoted by $\underline{t}$, and is called the component of *X* respect to $\underline{y}$. The approximation errors of *X* and $\underline{y}$ are defined as $E=X-\underline{t}{\underline{p}}^T$ and $f=\underline{y}-q\underline{t}$ respectively, where $\underline{p}$ is a *k* × 1 vector minimizing $\left|X-\underline{t}{\underline{p}}^T\right|$ and *q* is a scalar minimizing $\left|\underline{y}-q\underline{t}\right|$. Here $\underline{p}$ and *q* are called the loadings of $\underline{t}$ with respect to *X* and $\underline{y}$, respectively. This process can be repeated until the required halt condition is satisfied. A more detail description of the algorithm can be found in [42].

However, in real biological applications, linear relationships often fail to fully capture all the information among feature vectors extracted from biological data. Kernel methods project the data onto a high dimensional feature space to approach the problem, and are commonly used for revealing the intrinsic relationships hidden in the raw data. The kernel version of *pls* uses a nonlinear transformation Φ(.) to map the feature matrix into a higher-dimensional kernel space *K*, i.e., Φ : *x*_*i*_ ∈ *X*_*N* × *k*_ → Φ(*x*_*i*_) ∈ *K*. However, we only need to state the entire algorithm in terms of dot products between pairs of inputs and substitute the kernel function *K*(.,.) for it, instead of calculating the specific mathematical expression of nonlinear mapping. A detailed description of *kpls* can be found in [36].

### Description of the datasets

We conducted our experiments on three actual collections of metagenomes: two containing deep Illumina-based metagenomes, and one metagenomic dataset of low coverage sampled using 454 FLX Titanium technology. The first deep dataset was derived from the metagenomic project “A human gut microbial gene catalog established by deep metagenomic sequencing”, which was obtained from the faecal samples of 124 European individuals, and contains 25 IBD samples and 99 control samples [43]. The second deep dataset was derived from the metagenomic project “BGI Type 2 Diabetes study”, which was also obtained from the faecal samples, but from 145 Chinese individuals living in the south of China, and includes 71 T2D samples and 74 control samples [44]. The low coverage dataset was from the metagenomic study “Southampton Asthma metagenomics” which was obtained from both the sputum and the bronchoalveolar lavage samples of 55 individuals, and includes 66 asthma samples and 22 control samples [45]. The information of the three metagenomic datasets are detailed in the supplement (Additional file 1: Table S2).

### Verification experiment

Our work in this paper focuses on verifying the stability and generality of the DectICO algorithm, and comparing the classification performance of our proposed method with existing metagenomic sample classification methods. Therefore, we conducted two kinds of experiments, and defined them as stability test and generality test, in terms of differing purpose.

In the practical application of metagenomic sample classification, different researchers have usually sampled from different individuals for a specific disease. Consequently, multiple classifiers targeting the same disease will be trained by different samples. The similarity among the performance of classifiers reflects the stability of the metagenomic classification algorithm used. Therefore, we propose that the classification algorithm is considered stable, if the classifiers, which have been trained on a given kind of metagenomic data with different training sets, have similar classification performance. Our stability test was designed to verify the stability of a classification algorithm. Initially different groups of diseased and control samples are randomly selected from all of the samples 20 times with sample size, and then classifiers are trained based on these 20 training sets. Classification algorithm stabilities can be compared using the cross validation accuracies of the 20 classifiers.

The acquisition of diseased samples for a specific disease can be a limiting factor. The classifier trained by the samples labeled limited should distinguish all, or the major part of the unlabeled samples accurately. Namely, the classifiers trained by a classification algorithm should have good generality. Our generality test was designed to evaluate the generality of our method. Initially we select a group of the diseased and control samples randomly from all samples, and a classifier is trained by the training set. Next, 20 groups of the testing sets are selected of the same sample size randomly from the rest of the samples. The classification accuracies of these testing sets can then be obtained by the trained classifier. The generality of our proposed method can be assessed using the differences between the classification accuracies. The number of samples in the training and testing sets for the three metagenomic datasets is described in the Additional file 1 (Table S3).

Note that the numbers of the diseased and the control samples of the testing sets in our generality test are unbalanced (Additional file 1: Table S3). Therefore, we used the F1-measure to evaluate the classifying performance of the testing sets. The F1-measure is defined in the Additional file 1.

## Results and discussion

### DectICO performs better based on long oligonucleotides

**Fig. 2:**
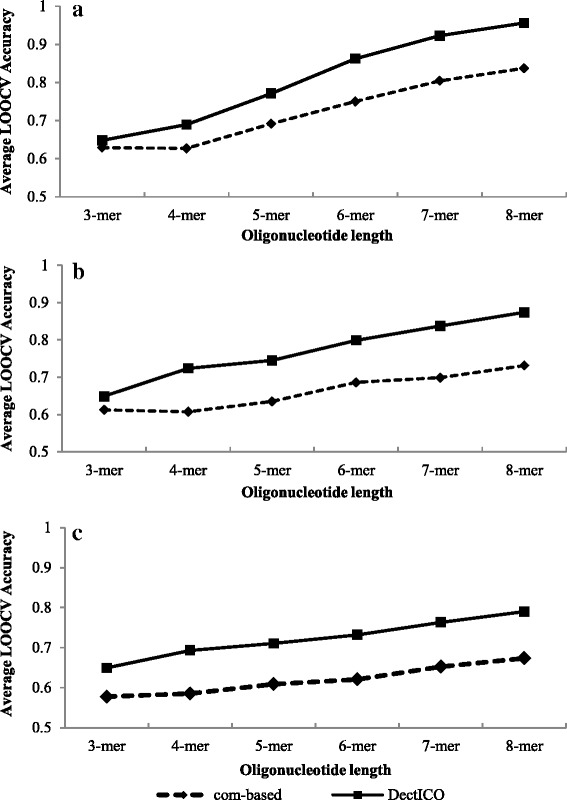
Comparison of LOOCV accuracies between DectICO and the sequence-composition-based method on the three metagenomic datasets. The average of LOOCV accuracies of the 20 classifiers trained in the stability test are compared between DectICO and the sequence-composition-based method based on the three collections of metagenomes. **a** corresponds to the asthma metagenomic samples. **b** and **c** correspond the IBD and T2D metagenomes, respectively. The solid lines with the square tags represent the classification performances of DectICO, while the dotted lines with the rhombic tags correspond to the sequence-composition-based method. The X-axis represents the length of the oligonucleotides, and the Y-axis corresponds to the average LOOCV accuracy

Figure 2 shows classification performance for the three metagenomic datasets obtained in our stability test. The average accuracy of the LOOCV procedure for the 20 trained classifiers using DectICO is compared with the sequence-composition-based method. The sequence features were extracted based on oligonucleotides with lengths varying from 3 to 8.

Primarily, for each kind of metagenome, we find the average LOOCV accuracies of DectICO increase as the length of oligonucleotide becomes longer. The accuracies of 8-mers are the highest among the varied lengths of oligonucleotides tested. Additionally, experimental results show that the average accuracies of DectICO are higher than the sequence-composition-based method for different lengths of oligonucleotides. Results illustrate the classifier trained by DectICO is more accurate for longer oligonucleotides, and also performs better than those trained by the sequence-composition-based method based on same length oligonucleotide. However, the difference in LOOCV accuracy between DectICO and the composition-based method for each classifier (See Additional file 2: Table S1) shows that DectICO does not outperform the composition-based method significantly for short oligonucleotides.

We also performed paired-sample *t*-test for the 20 groups of LOOCV accuracies of the classifiers between trained by DectICO and those trained by the sequence-composition-based method. The p-values of paired *t*-test were used to evaluate the statistical significance of the difference of classification performance between the two kinds of methods. Table 1 summarizes the p-values of paired *t*-test on the three kinds of metagenomic datasets. Results show that the p-values are less than 0.05 except for the 3-mer on the asthma dataset. Additionally, the p-values of paired *t*-test for the 3-mer and 4-mer are greater than based on the 7-mer and 8-mer in general. These results indicate that DectICO’s superior classification performance becomes more obvious as oligonucleotides get longer. Therefore, our experimental results illustrate that DectICO has better classification performance based on long oligonucleotides.

**Table 1:** *P*-values of paired *t*-test for the 20 groups of LOOCV accuracies between DectICO and the sequence-composition-based method

	3-mer	4-mer	5-mer	6-mer	7-mer	8-mer
Asthma	1.65E-01	4.18E-04	1.60E-05	1.36E-05	2.14E-05	7.42E-06
IBD	1.32E-02	1.57E-04	3.89E-08	5.93E-07	4.95E-11	1.42E-10
T2D	2.91E-06	7.33E-12	1.40E-09	1.10E-08	7.25E-07	1.76E-06

The paired-sample *t*-test for the 20 groups of LOOCV accuracies of the classifiers between those trained by DectICO and those trained by the sequence-composition-based method was also performed. And the p-values of paired *t*-test were used to evaluate the statistical significance of the difference of classification performance between the two kinds of methods

The above results all indicate that DectICO significantly outperforms the sequence-composition-based method based on long oligonucleotides. Apparently, the longer the oligonucleotide, the higher the dimensionality of the extracted sequence feature vector becomes, and the more information the sequence feature vector contains, from the metagenomic sample. Therefore, we conclude that the different diseased states of the samples are being represented sufficiently enough by the high-dimensional ICO vectors to give the classification high performance.

### DectICO is more stable and generalized than the sequence-composition-based method

We conducted our stability test on three actual metagenomic datasets to investigate DectICO’s stability. We then analyzed the difference in LOOCV accuracies among 20 trained classifiers for each kind of metagenome.

The LOOCV accuracy standard deviations for the 20 classifiers trained by DectICO and the sequence-composition-based method are presented in Table 2. This shows that the LOOCV accuracy standard deviations for the classifiers trained by DectICO are smaller than by the sequence-composition-based method, based on all different lengths of oligonucleotides, except for the 5-mer in the asthma dataset, the 3-mer in the IBD dataset, and the 3-mer and 4-mer in the T2D dataset. The standard deviations for DectICO range from 0.03 to 0.04 in the asthma and T2D datasets, while the standard deviations for the sequence-composition-based method are larger than 0.05 based on the 6-mer, 7-mer, and 8-mer. This result indicates that the difference in LOOCV accuracies among the 20 classifiers trained by DectICO is smaller than that by the sequence-composition-based method, especially based on long oligonucleotides. The classifiers trained by the different training sets with DectICO have more similar classification performances than those by the sequence-composition-based method. Therefore, we conclude our method is more stable than the sequence-composition-based algorithm.

**Table 2:** Comparison of the standard deviations of LOOCV accuracies for the 20 classifiers between DectICO and the sequence-composition-based method

		3-mer	4-mer	5-mer	6-mer	7-mer	8-mer
Asthma	com-based	0.057	0.064	0.061	0.068	0.069	0.063
	DectICO	0.053	0.048	0.065	0.033	0.033	0.031
IBD	com-based	0.045	0.081	0.065	0.056	0.034	0.042
	DectICO	0.063	0.057	0.048	0.041	0.031	0.027
T2D	com-based	0.041	0.026	0.034	0.039	0.057	0.051
	DectICO	0.048	0.028	0.027	0.037	0.038	0.034

We investigated DectICO’s stability using the results of stability test on three actual metagenomic datasets. The difference in LOOCV accuracies among 20 trained classifiers was analyzed with the LOOCV accuracy standard deviations for the 20 classifiers trained by DectICO and the sequence-composition-based method

We used the results of our generality test to compare the generality of DectICO and the sequence-composition-based algorithm. We also compared the standard deviations of the F1-measure for different testing sets between DectICO and the sequence-composition-based method (Table 3). Results show the standard deviations for DectICO range from 0.01 to 0.03, except for the 5-mer in the asthma dataset. However, the standard deviations corresponding to the sequence-composition-based method are all more than 0.03. The standard deviations for the sequence-composition-based method range from 0.047 to 0.054 for different lengths of oligonucleotides in the IBD dataset. These results demonstrate that the classification performance for different unlabeled samples with DectICO is more similar; that is, the DectICO algorithm is more generalized than the sequence-composition-based method.

**Table 3:** Comparison of the F1-measure standard deviations for the 20 testing sets between DectICO and the sequence-composition-based method

		3-mer	4-mer	5-mer	6-mer	7-mer	8-mer
Asthma	com-based	0.039	0.040	0.052	0.046	0.039	0.043
	DectICO	0.013	0.011	0.039	0.023	0.012	0.026
IBD	com-based	0.031	0.043	0.038	0.038	0.064	0.040
	DectICO	0.017	0.024	0.029	0.022	0.027	0.022
T2D	com-based	0.052	0.052	0.052	0.047	0.052	0.054
	DectICO	0.028	0.030	0.030	0.029	0.032	0.029

The generality of DectICO and the sequence-composition-based algorithm was compared with the standard deviations of the F1-measure for different testing sets in our generality test

The results above indicate that the classification performance of the DectICO algorithm is more similar for different training sets and for different testing sets. Therefore, DectICO is both more stable and more generalized than the sequence-composition-based method.

### Dynamic feature selection can promote the performance of the classifiers

As described in Algorithm, DectICO employs dynamic feature selection. Therefore, we also compared its classification performance against a non-dynamic feature-selection-based method using our stability test. The non-dynamic feature-selection-based method also employs the *kpls* feature selection algorithm and the ICO vectors.

**Fig. 3:**
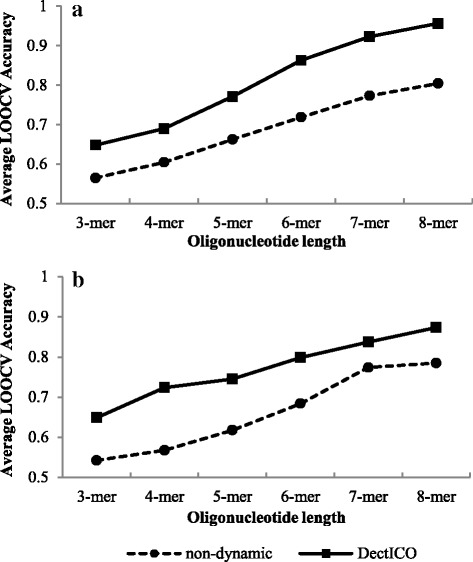
Comparison of classification performance between DectICO and the non-dynamic feature-selection-based method on the asthma and IBD datasets. The comparisons of the average LOOCV accuracies of the 20 classifiers between those trained by DectICO and those by the non-dynamic feature-selection-based method are presented. **a** and **b** correspond to the asthma and IBD metagenomes, respectively. The solid lines with the square tags represent the classification performances of DectICO, while the dotted lines with the rounded tags correspond to the non-dynamic feature-selection-based method. The framework of this figure is the same as Fig. 2

Figure 3 presents comparisons of the average LOOCV accuracies of the 20 classifiers between those trained by DectICO and those by the non-dynamic feature-selection-based method, on the asthma and IBD datasets. The results on the T2D dataset are shown in the Additional file 1 (Figure S2). We find that the average accuracies of the classifiers trained by DectICO are higher than those by the non-dynamic feature-selection-based method for different length oligonucleotides. The maximal difference of the average accuracies reaches 15 % for the 8-mer in the asthma dataset. Similar to Fig. 3, the classification performances for our method with T2D metagenomic dataset are also better than the non-dynamic feature-selection-based method (Additional file 1: Figure S2). Table 4 shows the p-values of paired *t*-test for the 20 groups of LOOCV accuracies of the classifiers between trained by DectICO and those trained by the non-dynamic feature-selection-based method on the three metagenomic datasets. Apparently, the p-values are all less than 0.05, which means the classification performances of DectICO are different from the non-dynamic feature-selection-based method significantly. That is, DectICO has an obvious superiority in classification compared to the non-dynamic feature-selection-based method.

**Table 4:** *P*-values of paired *t*-test for the 20 groups of LOOCV accuracies between DectICO and the non-dynamic feature-selection-based method

	3-mer	4-mer	5-mer	6-mer	7-mer	8-mer
Asthma	1.30E-09	1.37E-04	1.25E-05	2.29E-10	1.31E-08	3.41E-10
IBD	2.39E-12	2.31E-11	1.06E-10	1.59E-07	2.15E-04	5.17E-04
T2D	3.28E-07	1.16E-09	6.93E-10	3.70E-08	2.90E-08	2.83E-08

The results indicate that DectICO outperforms the non-dynamic feature-selection-based method. Therefore, we conclude that the dynamic feature selection method is more suitable for screening out useless information and noise in alignment-free metagenomic classification methods.

### DectICO outperforms the RSVM

Cui and Zhang recently proposed an alignment-free supervised metagenomic sample classification algorithm [37]. Their classification method employs the RSVM algorithm to perform feature selection and the classification is based on sequence composition. Consequently, we compared classification performance between DectICO and the RSVM-based algorithm for the three groups of metagenomic datasets. Because DectICO uses ICO vectors for classification, we also employed ICO vectors in the RSVM-based algorithm to make the comparison more reasonable.

**Fig. 4:**
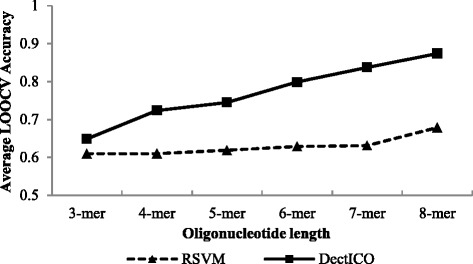
Comparison of classification performance between DectICO and RSVM based on the ICO with the IBD dataset. The solid lines with the square tags represent the classification performance of DectICO, while the dotted lines with the triangular tags correspond to the RSVM-based method. The framework of this figure is the same as Fig. 2

Figure 4 presents the average LOOCV accuracy of the 20 classifiers in our stability test trained by DectICO and RSVM with ICO vectors with the IBD dataset. Comparisons with the asthma and T2D datasets are shown in the Additional file 1 (Figure S3). We note that DectICO outperforms the RSVM-based method for all of the different length oligonucleotides tested with the IBD dataset. However, the classification superiority of DectICO with short oligonucleotides is less than it is for long oligonucleotides. The average LOOCV accuracy of our method is similar to the RSVM-based method with 3-mers. Additionally, comparisons with the asthma and T2D datasets (Additional file 1: Figure S2) show that the average LOOCV accuracies for DectICO are similar to those of RSVM for 3-mers and 4-mers. The difference in classification performance between DectICO and the RSVM-based method increases as the oligonucleotides become longer. The conclusion can also be derived from the p-values of the sample-paired *t*-test for the 20 groups of LOOCV accuracies of the classifiers between trained by DectICO and those trained by the RSVM-based method (Table 5). As shown in Table 5, the p-values for the 3-mer and 4-mer on the asthma dataset are 0.272 and 0.669 (>0.05) respectively, whereas the p-values are much less than 0.05 when the oligonucleotide length increases more than 6. Similar results can be obtained on the other two datasets. These results illustrate that DectICO outperforms the RSVM-based on ICO vectors method for long oligonucleotides more significantly so than for short oligonucleotides.

**Table 5:** *P*-values of paired *t*-test for the 20 groups of LOOCV accuracies between DectICO and the RSVM-based method

	3-mer	4-mer	5-mer	6-mer	7-mer	8-mer
Asthma	2.72E-01	6.69E-01	1.41E-02	2.91E-08	4.42E-11	2.26E-09
IBD	4.63E-02	3.02E-06	7.89E-08	5.25E-11	2.37E-10	3.13E-09
T2D	5.42E-01	6.27E-01	2.28E-02	4.59E-03	4.07E-05	1.34E-05

The stability and generality of DectICO were also compared with RSVM using our stability test and generality test. Table 6 presents the LOOCV accuracy standard deviations for the 20 classifiers using out stability test and the F1-measure standard deviations for the 20 testing sets using our generality test for DectICO and the RSVM-based method. The results in Table 6 only correspond to the IBD dataset; results for the asthma and T2D metagenomic datasets are shown in the Additional file 1 (Table S4 and S5).

**Table 6:** Comparison of the stability and generality between DectICO and the RSVM/ICO

	3-mer	4-mer	5-mer	6-mer	7-mer	8-mer
RSVM (stability test)	0.099	0.054	0.069	0.057	0.074	0.063
DectICO (stability test)	0.063	0.057	0.048	0.041	0.031	0.027
RSVM (generality test)	0.036	0.022	0.040	0.045	0.044	0.036
DectICO (generality test)	0.017	0.024	0.029	0.022	0.027	0.022

The stability and generality between DectICO and the RSVM/ICO are compared on the basis of the results of the stability test and generality test respectively

The LOOCV accuracy standard deviations for the 20 classifiers trained by DectICO are smaller than those for the RSVM-based method in general (Table 6), indicating that the performance of the classifiers trained by DectICO are more similar to each other than those by RSVM. Furthermore, the standard deviations of our method for 6-mers, 7-mers, and 8-mers range from 0.027 to 0.041 (Table 6, S3 and S4); however, the standard deviations corresponding to the RSVM-based method range from 0.057 to 0.09. Therefore, these results demonstrate that DectICO is more stable than RSVM, especially for long oligonucleotides. Similar situations occur in comparisons of generality between the two classification methods; DectICO outperforms RSVM again. However, the superiority of our method is not as significant as the stability, because the standard deviations of the RSVM-based method in our generality test are smaller than in the stability test, ranging from 0.022 to 0.059 (Table 6, Additional file 1: Table S3 and S4).

In summary, experimental results demonstrate that DectICO classifies metagenomic samples more accurately than the RSVM-based method with a set of completely labeled samples as training set, both with low and deep sequence depth metagenomic datasets. Additionally, our method is more stable and more generalized than the RSVM-based method.

## Conclusion

The alignment-free supervised classification method DectICO can accurately classify metagenomic samples without dependence on known microbial genomes. Selecting the ICO dynamically offers better stability and generality compared with sequence-composition-based classification algorithms. However, the metagenomic sample information obtained by the sequence features is limited. Subsequent work will focus on finding a hybrid feature combining sequence and functional features, also selected by *kpls*. Such a classification method is expected to have even better classification performance.

## Additional files

Additional file 1: Figure S1. Comparisons of classification performance between DectICO and the unsupervised alignment-free metagenomic clustering methods. **Table S2.** The information of the three collections of metagenomes. **Table S3.** The sizes of the training and testing sets for three collections of metagenomes used in our stability test and generality test. **Figure S2.** Comparisons of classification performances between DectICO and the non-dynamic feature-selection-based method on the T2D dataset. **Figure S3.** Comparisons of classification performances between DectICO and RSVM that based on the ICO on the asthma and T2D datasets. **Table S4.** Comparisons of the stability (from stability test) and generality (from generality test) between DectICO and the RSVM that with the ICO on asthma dataset. **Table S5.** Comparisons of the stability (from stability test) and generality (from generality test) between DectICO and the RSVM that with the ICO on T2D dataset. **Table S6.** The runtime of calculation for the feature vectors of ICO based on three kinds of metagenomic samples and 1 Mbp contig. **Table S7.** The consumed RAM of calculation for the feature vectors of ICO based on three kinds of metagenomic samples. **Table S8.** The runtime of classification process with varying rounds of feature selection and different numbers of samples in training set on the T2D metagenomes. **Table S9.** The consumed RAM of classification process with varying rounds of feature selection and different numbers of samples in training set on the T2D metagenomes. **Table S10.** The ICO vector dimension for different length oligonucleotides. (DOCX 76 kb)
Additional file 2: Table S1. The difference of the LOOCV accuracy between DectICO and the-sequence-composition-based method for each classifier. (XLSX 13 kb)

## Abbreviations

SVM: Support vector machine
NIH: National Institutes of Health
OTU: Operational taxonomic unit
WMGS: Whole metagenome shotgun sequencing
RDP: Ribosomal database project
KEGG: Kyoto Encyclopedia of Genes and Genomes
COG: Cluster of Orthologous Groups of proteins
NCBI: National center for biotechnology information
ICO: Intrinsic correlation of oligonucleotides
IBD: Inflammatory bowel disease
T2D: Type 2 diabetes
kpls: Kernel partial least squares
RSVM: Recursive support vector machine
LOOCV: Leave-one-out cross validation
EMBL: European Molecular Biology Laboratory
