# Supplementary material for: DectICO: an alignment-free supervised metagenomic classification method based on feature extraction and dynamic selection
Source: BMC Bioinformatics. 2015 Oct 7;16:323. doi: 10.1186/s12859-015-0753-3 (PMC4596415; doi:10.1186/s12859-015-0753-3)
Supplement: Additional file 1: Figure S1. — Comparisons of classification performance between DectICO and the unsupervised alignment-free metagenomic clustering methods. Table S2. The information of the three collections of metagenomes. Table S3. The sizes of the training and testing sets for three collections of metagenomes used in our stability test and generality test. Figure S2. Comparisons of classification performances between DectICO and the non-dynamic feature-selection-based method on the T2D dataset. Figure S3. Comparisons of classification performances between DectICO and RSVM that based on the ICO on the asthma and T2D datasets. Table S4. Comparisons of the stability (from stability test) and generality (from generality test) between DectICO and the RSVM that with the ICO on asthma dataset. Table S5. Comparisons of the stability (from stability test) and generality (from generality test) between DectICO and the RSVM that with the ICO on T2D dataset. Table S6. The runtime of calculation for the feature vectors of ICO based on three kinds of metagenomic samples and 1 Mbp contig. Table S7. The consumed RAM of calculation for the feature vectors of ICO based on three kinds of metagenomic samples. Table S8. The runtime of classification process with varying rounds of feature selection and different numbers of samples in training set on the T2D metagenomes. Table S9. The consumed RAM of classification process with varying rounds of feature selection and different numbers of samples in training set on the T2D metagenomes. Table S10. The ICO vector dimension for different length oligonucleotides. (DOCX 76 kb) [file 12859_2015_753_MOESM1_ESM.docx]

**The comparisons of classification performance between DectICO and the unsupervised alignment-free metagenomic clustering methods**

**Methods**

The method proposed in “Comparison of metatranscriptomic samples based on *k*-Tuple Frequencies” employs different dissimilarity measures (three $d_{2}-type$ dissimilarity measures, one dissimilarity measure in CVTree, one relative entropy based measure $S2$and three classical $l_{p}-norm$ distances) and the hierarchical clustering algorithm to compare the metatranscriptomic or metagenomic samples. These dissimilarity measures calculated based on the *k*-mer frequencies. For the comparison of genomes or long sequences, one widely used statistic is *D*_2_, which is a correlation between the frequencies of k-mers for two sequences of interest [[1](#_ENREF_1)]. However, it was shown that *D*_2_ was dominated by the noise caused by the randomness of the sequences and has low statistical power to extract the potential relationship between two sequences [[2](#_ENREF_2)]. A new statistic$D_{2}^{S}$ was developed by standardizing the k-mers counts with their means and standard deviations [[3](#_ENREF_3), [4](#_ENREF_4)].$D_{2}^{S}$ is more powerful than the *D*_2_ statistic for the detection of relationships between sequences related through a common motif model that the two sequences share instances of one or multiple motifs. $d_{2}^{S}$was further normalized by$D_{2}^{S}$, with a range from 0 to 1, in order to reduce the effects of sequence length [[5](#_ENREF_5)]. Based on the experimental results in many researches, $d_{2}^{S}$ has best performances for distinguishing sequences and clustering metagenomic samples. Therefore, we chose the$d_{2}^{S}$for the comparisons in the supplemental experiment [[5](#_ENREF_5), [6](#_ENREF_6)].

Therefore, we also employed the $d_{2}^{S}$ dissimilarity measure with the varying orders of the Markov model (0, 1, 2 and 3) and the different lengths of *k*-mer (range from 3 to 8) in our experiment. Because the hierarchical clustering algorithm can’t obtain quantitative results of clustering, we employed the spectral clustering algorithm to finish the comparisons. Spectral clustering is a graphic theory based clustering algorithm, which performs the clustering by dividing a weighted undirected graph into multiple sub-graphs. The spectral clustering algorithm employs the method of graph cutting to resolve the problem of locally optimal solution. Therefore, compared with “traditional algorithms”, such as k-means, the spectral clustering can obtain higher clustering accuracy. Additionally, the spectral clustering algorithm uses the laplacian eigenmap to reduce the dimension of the original feature vectors in order to reduce the computation complexity of the clustering algorithm[[7](#_ENREF_7)].

The asthma and T2D metagenomic datasets were used in our experiments. We chose ten testing sets generated in generality test for each kind of metagenome, and classified the ten groups of testing sets with DectICO and the unsupervised alignment-free method.

**Results and discussion**

Figure S1 presents the average classification accuracies of the ten testing sets with the two kinds of classification methods. F1-measure was also used to evaluate the performances of DectICO. However, the unsupervised alignment-free methods used the general accuracy, because there is no F1-measure for clustering algorithm. The results obviously show that DectICO outperforms the unsupervised alignment-free methods except for the 3-mer on the asthma dataset. In addition, the classification performances of our method are better than the unsupervised alignment-free methods significantly on the T2D metagenomes.

**Figure S1.** **Comparisons of classification performances between DectICO and the unsupervised alignment-free methods on the asthma and T2D datasets.**

(a) and (b) correspond to the asthma and T2D metagenomes respectively. The solid lines with the square tags represent the classification performances of DectICO while the dotted lines with different tags correspond to the unsupervised alignment-free methods. Different tags mean different orders of the Markov model.

Further study shows that the performances of the unsupervised alignment-free methods on the asthma dataset are better than on the T2D dataset. Most of the average accuracies of the unsupervised alignment-free methods on the T2D metagenomes are lower than 60%. As described in the introduction of our manuscript, the unsupervised alignment-free methods can only discover major intrinsic clustering relations among the compared samples. Therefore, we suppose that the T2D diseased and the health status are not the most powerful clustering relations for these samples. As a supervised alignment-free method, our method extracts the intrinsic difference between the samples with different statuses based on the training sets, classifying the unlabeled samples accurately.

The ICO vector dimension for different length oligonucleotide

**The information of the three collections of metagenomes**

The three kinds of metagenomic sequencing datasets were downloaded from the EMBL database, and the information of the three collections of metagenomes is showed in Table S2

**Table S2**. The information of the three collections of metagenomes.

| STUDY_ID | PROJECT_NAME | NUMBER_OF_SAMPLES | CENTRE_NAME |
| --- | --- | --- | --- |
| ERP000108 | A human gut microbial gene catalog established by deep metagenomic sequencing | 124 | BGI |
| SRP008047 | BGI Type 2 Diabetes study | 145 | BGI |
| ERP006003 | Southampton Asthma metagenomics | 88 | Inflammation |

**The sizes of the training and testing sets for three kinds of metagenomes**

Table S3 presents the sizes of the training and testing sets for the three metagenomic datasets in stability test and generality test experiments. For the IBD samples, we randomly selected 20 diseased samples and 20 control samples twenty times to compose the twenty training sets in the stability test. With regards to the generality test, we first chose the 15 diseased and 15 control samples randomly to obtain the training set, and then selected 8 diseased samples and 50 control samples twenty times from the rest samples to get the twenty testing sets.

For the T2D samples, 40 diseased samples and 40 control samples were chose twenty times randomly in the stability test. And the training set in the generality test also consisted of 40 diseased samples and 40 control samples which were selected randomly. The twenty testing sets contained 20 diseased and 20 control samples were also chose from the rest samples randomly.

For the asthma samples, 12 diseased samples and 12 control samples were chose twenty times randomly in the stability test. And the training set in the generality test consisted of 9 diseased samples and 9 control samples which were selected randomly. Then we selected 20 diseased and 5 control samples twenty times from the rest samples randomly to compose the twenty testing sets.

**Table S3**. The sizes of the training and testing sets for the three collections of metagenomes in stability test and generality test.

|  | | stability test | | | generality test | | |
| --- | --- | --- | --- | --- | --- | --- | --- |
|  |  | IBD | T2D | asthma | IBD | T2D | asthma |
| Training set | Diseased sample | 20 | 40 | 12 | 15 | 40 | 9 |
|  | Control sample | 20 | 40 | 12 | 15 | 40 | 9 |
| Testing set | Diseased sample |  |  |  | 8 | 20 | 20 |
|  | Control sample |  |  |  | 50 | 20 | 5 |

**The definition of the F1-measure**

The definition of the F1-measure is described as follows:

$$F1=2*\frac{precision*recall}{precision+recall}$$

$$precision=\frac{tp}{tp+fp}$$

$$recall=\frac{tp}{tp+fn}$$

where $tp$,$fp$ and $fn$ represent the number of true positive, the number of false positive and the number of false negative respectively.

**Comparisons of classification performances between DectICO and the non-dynamic feature selection based method**

**Figure S2. Comparisons of classification performances between DectICO and the non-dynamic feature selection based method on the T2D dataset.**

The solid lines with the square tags represent the classification performances of DectICO while the dotted lines with the rounded tags correspond to the non-dynamic feature selection based method.

**Comparisons of classification performances between DectICO and the RSVM based method**

**Figure S3. Comparisons of classification performances between DectICO and RSVM that based on the ICO on the asthma and T2D datasets.**

(a) and (b) correspond to the asthma and T2D metagenomes respectively. The solid lines with the square tags represent the classification performances of DectICO while the dotted lines with the triangular tags correspond to the RSVM based method.

**Table S4.** Comparisons of the stability (from stability test) and generality (from generality test) between DectICO and the RSVM that with the ICO on asthma dataset.

|  | 3-mer | 4-mer | 5-mer | 6-mer | 7-mer | 8-mer |
| --- | --- | --- | --- | --- | --- | --- |
| RSVM (stability test) | 0.084 | 0.075 | 0.106 | 0.083 | 0.080 | 0.090 |
| DectICO (stability test) | 0.053 | 0.048 | 0.065 | 0.033 | 0.033 | 0.031 |
| RSVM (generality test) | 0.044 | 0.048 | 0.044 | 0.039 | 0.046 | 0.052 |
| DectICO (generality test) | 0.013 | 0.011 | 0.039 | 0.023 | 0.012 | 0.026 |

**Table S5.** Comparisons of the stability (from stability test) and generality (from generality test) between DectICO and the RSVM that with the ICO on T2D dataset.

|  | 3-mer | 4-mer | 5-mer | 6-mer | 7-mer | 8-mer |
| --- | --- | --- | --- | --- | --- | --- |
| RSVM (stability test) | 0.058 | 0.049 | 0.063 | 0.068 | 0.067 | 0.062 |
| DectICO (stability test) | 0.048 | 0.028 | 0.027 | 0.037 | 0.038 | 0.034 |
| RSVM (generality test) | 0.056 | 0.057 | 0.051 | 0.059 | 0.057 | 0.053 |
| DectICO (generality test) | 0.028 | 0.030 | 0.030 | 0.029 | 0.032 | 0.029 |

**The runtime and required RAM of DectICO**

Although DectICO isn’t characterized by fast and low RAM consumed, we evaluated the DectICO algorithm in term of the time and space complexity. In addition, we also investigate the runtime and required RAM of our algorithm on actual metagenomic sample classification. Evaluation was performed on a workstation with CPU Intel Xeon E5-2665 (2.4 GHz, 32 processors) and 32GB RAM installed.

The algorithm of DectICO is primarily divided into two steps: calculate the feature vectors of metagenomic samples and classify the samples with the selected feature sets. As shown in Table S6~S9, the process of calculating the vector of ICO cost primary runtime. Therefore, we evaluated the time complexity of this process. Therefore, we evaluated the time complexity of this process. Based on the principle of ICO, there are two parts within the ICO vector. The time complexity of calculating the two parts are both$O(4^{n})$ , where *n* represents the length of oligonucleotide used for extracting the ICO. Therefore the overall time complexity of calculation process of ICO is:$T(n)=O(4^{n})$. It is shown that with the length of oligonucleotide increasing, the exponential growth in runtime will make the DectICO algorithm time-consuming. However, this is a common problem for alignment-free methods. Compared with DectICO, the RSVM based classification method uses the frequency of *k*-mer as the sequence feature. The time complexity of calculating the frequency vector is:$T(m)=O(4^{m})$ , where *m* represents the length of *k*-mer, indicating that the runtime also has the exponential growth when m increases. On the other hand, the space complexity of the algorithm for calculating the ICO vector is:$S(n)=O(4^{n})$, where *n* also represents the length of oligonucleotide used for extracting the ICO, the same as the feature vector calculation step in RSVM based algorithm. Therefore, our algorithm has similar performance to the RSVM based algorithm in term of the time complexity and space complexity.

We also gather a statistics of the runtime and required RAM of DectICO for actual metagenomic classification. Table S6 and S7 show the runtime and required RAM for calculating the feature vector of ICO based on three kinds of metagenomic samples respectively. Firstly, the consumed RAM of calculations (Table S7) are all very low, the highest consumed RAM is only 51 MB. As shown in Table S6, the calculation of the ICO vector of 8-mer for 1 M-bp contig only cost about 68 seconds, the cost time is acceptable. Because the IBD dataset is very large, the runtime of the calculation of ICO vector for 8-mer on the IBD metagenomes is the longest, reaches 701151 seconds (about 8 days). Considering that the calculation of the ICO vector for metagenomes is a one-off process, this runtime is acceptable. In summary, the runtime and consumed RAM of calculating the ICO vector of metagenomic samples are both reasonable.

**Table S6.** The runtime (second) of calculation for the feature vectors of ICO based on three kinds of metagenomic samples and 1 Mbp contig.

|  | 3-mer | 4-mer | 5-mer | 6-mer | 7-mer | 8-mer |
| --- | --- | --- | --- | --- | --- | --- |
| 1 M-bp  Asthma | 14  3485 | 23  5584 | 33  7841 | 42  10165 | 52  12631 | 68  16360 |
| IBD | 149353 | 239331 | 336065 | 435667 | 541359 | 701151 |
| T2D | 123990 | 198688 | 278994 | 361682 | 449426 | 582082 |

**Table S7.** The consumed RAM (MB) of calculation for the feature vectors of ICO based on three kinds of metagenomic samples.

|  | 3-mer | 4-mer | 5-mer | 6-mer | 7-mer | 8-mer |
| --- | --- | --- | --- | --- | --- | --- |
| Asthma | 42~46 | 42~48 | 42~48 | 42~49 | 39~46 | 41~50 |
| IBD | 41~46 | 42~49 | 44~50 | 40~46 | 42~49 | 42~48 |
| T2D | 42~47 | 40~48 | 42~49 | 41~50 | 44~51 | 40~49 |

*Note:* The consumed RAM is within a range.

The runtime and consumed RAM of the process of classification with the selected feature sets were also evaluated. Based on the principle of classification, the runtime and consumed RAM depend on the round of feature selection and the number of samples in training set. Therefore, we evaluated the runtime and required RAM of classification process based on varying rounds of feature selection and different numbers of samples in training set. Table S8 and S8 show the runtime and consumed RAM of classification with the selected feature sets respectively. The round of feature selection increases as the oligonucleotide becomes longer, because the dimension of the ICO vector for long oligonucleotide is higher than for short oligonucleotide. The results in Table S8 show that the runtime of all classification processes are short, the longest runtime is 916 seconds, only about 15 minutes (17 rounds of feature selection and 100 samples in training set). And the runtimes for 3-mer (4 rounds of feature selection) are all about 1 second. Table S9 shows that the consumed RAM are within in the range of 400 to 450 MB. The required RAM is also acceptable.

**Table S8.** The runtime of classification process with varying rounds of feature selection and different numbers of samples in training set on the T2D metagenomes.

|  | 3-mer | 4-mer | 5-mer | 6-mer | 7-mer | 8-mer |
| --- | --- | --- | --- | --- | --- | --- |
| Round of feature selection (time) | 4 | 7 | 10 | 12 | 16 | 17 |
| 20 samples (second) | 1 | 2 | 6 | 14 | 80 | 232 |
| 40 samples (second) | 1 | 2 | 9 | 23 | 136 | 315 |
| 60 samples (second) | 1 | 3 | 11 | 34 | 224 | 587 |
| 80 samples (second) | 1 | 5 | 14 | 53 | 357 | 748 |
| 100 samples (second) | 2 | 6 | 22 | 79 | 558 | 916 |

**Table S9.** The consumed RAM of classification process with varying rounds of feature selection and different numbers of samples in training set on the T2D metagenomes.

|  | 3-mer | 4-mer | 5-mer | 6-mer | 7-mer | 8-mer |
| --- | --- | --- | --- | --- | --- | --- |
| Round of feature selection (time) | 4 | 7 | 10 | 12 | 16 | 17 |
| 20 samples (MB) | 398 | 399 | 399 | 397 | 409 | 424 |
| 40 samples (MB) | 399 | 400 | 400 | 403 | 421 | 429 |
| 60 samples (MB) | 401 | 401 | 400 | 399 | 422 | 430 |
| 80 samples (MB) | 400 | 399 | 401 | 404 | 431 | 434 |
| 100 samples (MB) | 402 | 400 | 403 | 409 | 439 | 446 |

*Note:* Because some classification processes are too short to record the change of RAM, we only show the max consumed RAM in the classification processes.

**The ICO vector dimension for different length oligonucleotides**

**Table S10.** The ICO vector dimension for different length oligonucleotides.

|  | 3-mer | 4-mer | 5-mer | 6-mer | 7-mer | 8-mer |
| --- | --- | --- | --- | --- | --- | --- |
| Dimension of ICO vector | 84 | 476 | 2388 | 11636 | 54612 | 251348 |

**References**

1. Blaisdell BE: **A measure of the similarity of sets of sequences not requiring sequence alignment**. *Proceedings of the National Academy of Sciences of the United States of America* 1986, **83**(14):5155-5159.

2. Lippert RA, Huang H, Waterman MS: **Distributional regimes for the number of k-word matches between two random sequences**. *Proceedings of the National Academy of Sciences of the United States of America* 2002, **99**(22):13980-13989.

3. Reinert G, Chew D, Sun F, Waterman MS: **Alignment-free sequence comparison (I): statistics and power**. *Journal of computational biology : a journal of computational molecular cell biology* 2009, **16**(12):1615-1634.

4. Wan L, Reinert G, Sun F, Waterman MS: **Alignment-free sequence comparison (II): theoretical power of comparison statistics**. *Journal of computational biology : a journal of computational molecular cell biology* 2010, **17**(11):1467-1490.

5. Song K, Ren J, Zhai Z, Liu X, Deng M, Sun F: **Alignment-free sequence comparison based on next-generation sequencing reads**. *Journal of computational biology : a journal of computational molecular cell biology* 2013, **20**(2):64-79.

6. Wang Y, Liu L, Chen L, Chen T, Sun F: **Comparison of metatranscriptomic samples based on k-tuple frequencies**. *PloS one* 2014, **9**(1):e84348.

7. Chen WY, Song Y, Bai H, Lin CJ, Chang EY: **Parallel spectral clustering in distributed systems**. *IEEE transactions on pattern analysis and machine intelligence* 2011, **33**(3):568-586.
